# Reducing the Risk of Healthcare Associated Infections from *Legionella* and Other Waterborne Pathogens Using a Water Management for Construction (WMC) Infection Control Risk Assessment (ICRA) Tool

**DOI:** 10.3390/idr14030039

**Published:** 2022-05-06

**Authors:** Molly M. Scanlon, James L. Gordon, Angela A. Tonozzi, Stephanie C. Griffin

**Affiliations:** 1 Standards and Research, Phigenics, LLC, 3S701 West Avenue, Suite 100, Warrenville, IL 60555, USA; 2 Department of Community, Environment and Policy, Mel and Enid Zuckerman College of Public Health, University of Arizona, Tucson, AZ 85724, USA; scgriffin@arizona.edu; 3 Gordon Architectural Design, Coronado, CA 92118, USA; jimgordonarchitect@yahoo.com; 4 Angela A. Tonozzi, Physician Consultant, Milwaukee, WI 53217, USA; atonozzi@gmail.com

**Keywords:** construction, healthcare associated infections, infection control, *Legionella*, risk assessment, waterborne pathogen, water management, water safety plan

## Abstract

Construction activities in healthcare settings potentially expose building occupants to waterborne pathogens including *Legionella* and have been associated with morbidity and mortality. A Water Management for Construction—Infection Control Risk Assessment (WMC-ICRA) tool was developed addressing gaps in building water management programs. This enables healthcare organizations to meet the requirements of ANSI/ASHRAE Standard 188 referenced in numerous guidelines and regulations. A WMC-ICRA was modeled after the ICRA required for prevention and control of airborne pathogens to reduce the risk of healthcare associated infections. The tool allows users to evaluate risk from waterborne pathogen exposure by analyzing construction activities by project category and building occupant risk group. The users then select an appropriate level of risk mitigation measures. Technical aspects (e.g., water age/stagnation, flushing, filtration, disinfection, validation testing), are presented to assist with implementation. An exemplar WMC-ICRA tool is presented as ready for implementation by infection prevention and allied professionals, addressing current gaps in water management, morbidity/mortality risk, and regulatory compliance. To reduce exposure to waterborne pathogens in healthcare settings and improve regulatory compliance, organizations should examine the WMC-ICRA tool, customize it for organization-specific needs, while formulating an organizational policy to implement during all construction activities.

## 1. Introduction

Construction activities are a known risk factor contributing to disease cases and deaths in community and healthcare settings from waterborne pathogen growth and spread in a building water distribution system (BWDS) [1,2,3]. Construction activity risk factors associated with the BWDS include excavation, re-pressurization, demolition activities, efficiency design, underground utility connections, construction equipment with water reservoirs, water main breaks, vibration activities, and commissioning the building water system [1]. Public health officials and healthcare providers have reported disease cases (*n* = 894) and deaths (*n* = 112) from waterborne pathogens associated with construction activities from events dating back to 1965 and summarized through 2016 [1]. These events are likely underreported. The lack of appropriate commissioning activities in a building water system prior to occupancy is the highest construction activity risk factor resulting in disease cases (*n* = 472) and deaths (*n* = 68) [1].

Construction activities encompass a wide scope of work from minor maintenance and repair projects to the assembly of an entire new building [4]. In healthcare facilities utility systems require careful 24-h monitoring inclusive of construction activities to avoid obvious life-safety impacts to patients receiving medical treatment [5,6]. Since the early 2000’s healthcare codes, standards, and best practices have included reducing the risk of healthcare associated infections (HAIs) from air and waterborne pathogens associated with construction activities [7]. As regulatory requirements have advanced, authorities having jurisdiction (AHJ) emphasize and enforce the implementation of an infection control risk assessment (ICRA) for construction activities to reduce HAIs. A construction ICRA is a series of processes for project-specific continuous assessment which will implement interventions, monitoring, and improvement as an organizational program to protect patients, staff, and visitors during projects for construction, renovation, and maintenance and repair [8].

The construction ICRA framework initially focused on airborne pathogens, namely *Aspergillus*, emerging from mold spores from gypsum board and demolition activities [4,7,9,10]. Waterborne pathogens including *Legionella* are known to be associated with construction activities [1,2]. However, construction ICRA tools have not expressly included: (1) a system of water hazard analysis; (2) the application of hazard controls associated with waterborne pathogens for construction activities; or (3) any discussion of methods for validation testing for water quality and safety [8,11]. Prior to 2015 waterborne pathogen guidance documents emphasized aggressive mitigation measures (i.e., chemical treatment systems, thermal heating, or hyperchlorination) as a rapid intervention for waterborne pathogens (e.g., *Legionella*) [12]. These control methods are still applicable under specific circumstances [13]. However, these rapid interventions are no longer recommended for implementation outside of a total system of risk management for the prevention of waterborne pathogens in building water systems [2,13,14]. The risk management system for waterborne pathogens in the US is commonly referred to as a building water management program (WMP) and internationally this is referred to as a water safety plan [3,14]. Although there has been some regulatory guidance on commissioning building water systems [14], there are minimal tools available to guide the over 6000 US healthcare providers and 14,000 US skilled nursing facilities through a risk assessment process to subsequently control waterborne pathogen growth and spread in building water systems from construction activities [14,15,16,17]. The purpose of this manuscript is to present a water management for construction (WMC) ICRA tool. The tool is intended to address the gap between the practice of water management in healthcare settings and the requirement of implementing an ICRA inclusive of mitigation for waterborne pathogen growth and spread associated with construction activities. The WMC-ICRA tool presented herein acts as a framework to assist healthcare organizations with alignment to other design and construction industry policies, standards, and building codes for healthcare settings to reduce HAIs from waterborne pathogens.

## 2. Background/Literature Review

Through CDC investigations, it has been demonstrated that implementation of a WMP inclusive of risk from construction activities is a viable process to reduce the likelihood of illness, injury, and death from waterborne pathogens [2]. A WMP is currently defined as “the risk management plan for the prevention and control of Legionellosis associated with building water systems, including documentation of the plan’s implementation and operation” [14] (p. 3). Additional standards are under development to expand this WMP definition to include other pathogens of interest beyond *Legionella* [18]. With the publication and agency enforcement of ANSI/ASHRAE Standard 188: Legionellosis: Risk Management for Building Water Systems, it is imperative that construction risk factors be considered within a comprehensive WMP. Per Section 4.2.1, Building Owner Requirements, the owner must analyze any new building, renovation, addition, or modification to an existing building and its water systems for the risk of *Legionella* growth and spread in building water systems [14]. For renovation projects, this analysis is to occur before construction begins [14]. For newly constructed buildings, this is to occur prior to official occupancy of the building and use for patient care operations. Additionally, Section 8.4, Commissioning, requires a commissioning plan be incorporated into the project as part of the requirements for activating building water systems [14].

Similarly, the National Academies of Sciences, Engineering, and Medicine (NASEM) 2020 publication, Management of *Legionella* in Water Systems, recommends a project specific design and commissioning plan for *Legionella* control be prepared for the construction project [19]. The NASEM (2020) report recommended the building design initiate the WMP to address commissioning for *Legionella* control including verifying the absence of *Legionella* in premise plumbing before occupancy. However, there has been minimal, if any guidance to effectively engage key stakeholders especially the planning, design, and construction industries in the water management process [1,19]. There is an imbalance within the design and construction industries focusing on water efficiency and conservation, while minimal efforts are undertaken to deal with water safety regulatory requirements for building occupants [19]. Meanwhile, healthcare building owners are expected to have water systems safe and properly managed upon first day of patient care operations [14,20].

In the US it is common practice, and in many cases a legal requirement, to perform an ICRA prior to the start of any construction activities in both existing and new healthcare buildings [4,5,8,21]. A healthcare construction ICRA for airborne pathogens utilizes a standard decision-making matrix by defining: (1) project activity types, (2) impacted patient risk groups, and (3) class of precaution groupings (i.e., a group of mitigation measures) for implementation [8] (Chapter 2).

An ICRA framework was introduced into the healthcare construction industry as a best practice starting in the early 2000’s [7]. It has subsequently evolved into a project requirement within the Facility Guideline Institute’s 2018 Guideline for the Design and Construction of Hospitals (commonly known as the FGI Guidelines) [5]. A construction ICRA for airborne pathogens is also endorsed by The Joint Commission (TJC) and the Association of Professionals for Infection Control and Epidemiology (APIC) [8,22]. Since its inception healthcare construction ICRA practices intended to address both air and waterborne pathogens [7]. As an early adopter of water safety programs, The Veterans Health Administration (VHA) Directive 1061, Prevention of Health Care-Associated *Legionella* Disease and Scald Injury from Water Systems, states that an “ICRA is conducted in cooperation with other VA medical facility stakeholders, to address the potential impact of construction and maintenance of water systems on growth or transmission of waterborne pathogens and to determine the extent of precautions, disinfection and system or component commissioning requirements” [23] (p. 16).

Healthcare settings are highly controlled environments with numerous protocols, methods, and best practices enforced through multiple agencies for the control of utility and engineering systems [21]. These utility systems (mechanical, electrical, and plumbing) require monitoring for on-going operations and prevention of any disruption or failure of utility systems in and around patient care environments [5]. In the US, establishing standards for healthcare design and construction began in 1947 with the General Standards for the Hill-Burton Act [9]. These guidelines for over 75 years have evolved into today’s FGI Guidelines as consensus documents which are frequently adopted by reference into law as the minimum standards for the design and construction of healthcare buildings by US federal and state AHJ [4]. Currently, the FGI Guidelines require the implementation of a construction ICRA for the protection of air and waterborne pathogens [5]. The use of an ICRA process matured out of continually emerging evidence-based research linking disease cases and deaths to HAIs associated with construction activities [7]. Although airborne and waterborne transmission have been equally mentioned since inception of the ICRA construction framework, current construction ICRA policies in hospitals primarily address airborne pathogens (e.g., *Aspergillus*) with minimal if any guidance for waterborne pathogens (e.g., *Legionella*) [11]. If infection control risk mitigation recommendations (ICRMR) for water are mentioned, they tend to discuss mold abatement from water leakage, flooding, and damage to surrounding building materials [8].

The leading guidance document, ANSI/ASHRAE Standard 188, Section 8 addresses the design and commissioning of the building’s water system but does not explicitly address construction activity risk factors and mitigation measures [14]. Commissioning procedures such as flushing and disinfection are required to occur within a three-week time frame prior to beneficial occupancy [14]. Additionally, ANSI/ASHRAE Standard 188 Annex A specifically mentions healthcare facilities need to address updating WMPs as the building changes over time through renovation and expansion [14]. Annex A states the healthcare organizations are to “conduct an evaluation and estimate of the likelihood of Legionellosis risk as necessary for the project: (1) during the early planning; (2) during each phase of design and construction; and (3) during commissioning” [14] (p. 13). This echoes the NASEM (2020) report recommendation to initiate the WMP planning during design and move toward implementation during construction and commissioning [19]. This leads to more effective water quality and safety management during on-going operations. Risk assessments are to be conducted on a project-by-project basis; however, no specific tools currently exist on how to effectively implement any of this as part of a building owner’s healthcare construction program.

As a companion document, ASHRAE Guideline 12-2020: Managing the Risk of Legionellosis Associated with Building Water Systems states repaired and recently constructed and commissioned building water systems are at increased risk of *Legionella* colonization [13]. The document emphasizes the need for appropriate planning, design, and construction of potable and non-potable building water systems. Even though commissioning activities often describe flushing, cleaning, or disinfection methods of the building water system before occupancy [14], the guidance document [13] does not give adequate direction to navigate the myriad of construction project types or to help stakeholders identify construction activity risk factors.

In summary, present guidance documents are not formulated to address the ICRA framework [8] and its corresponding ICRMRs for building water systems paralleling what is common practice for mechanical air ventilation [11,21]. This makes advancing the healthcare provider’s comprehensive WMP policy and procedures inclusive of construction activities challenging. To advance the application of WMPs the current authors propose developing a water management for construction (WMC) ICRA tool, as a parallel framework and first step to address the gap for incorporating water management practices into healthcare construction policies and procedures. To the authors’ knowledge there are minimal tools or guidance documents for building owners suggesting how to evaluate the magnitude, scale, or scope of construction activities affecting building water systems and the subsequent commissioning of water quality and water safety of the potable water systems for healthcare facilities.

## 3. Materials and Methods

The proposed WMC-ICRA tool will leverage core principles of an established construction-based ICRA framework [8] and established WMP implementation methods for water quality and safety [13,14,24,25]. The intent is to create alignment with existing construction standards and practice to further the development of a comprehensive WMP. The methods stated herein will outline and provide a new novel decision matrix for healthcare facilities for water management during construction practices for risk assessment for a BWDS.

### 3.1. Developing WMC-ICRA Project Types

The ICRA framework will be adjusted for water by modifying the construction project types (A, B, C, and D). The key parameters involve including principles of water age [13] and defining the scope of construction activities [8] related to water system component changes (repaired, maintained, modified, or newly constructed).

#### 3.1.1. Adjusting for Water Age/Dormancy

Water age is a significant risk factor contributing to the growth and spread of waterborne pathogens in building water systems [13,24]. During construction high water age occurs when minimal water is used from the time the building water system is activated (i.e., filling the piping with water throughout the distribution system) and until the building is used for normal operations [13]. Within the ICRA framework, water age (time of dormancy) would need to align with Project Types A, B, C, and D from low, non-invasive activities to major disruptive activities.

#### 3.1.2. Defining Construction Scope of Work for Water System Components

Construction scope category definitions for A, B, C, and D will be modified to include a definition for level of BWDS components scheduled for repair, replacement, or new installation. Plumbing and BWDS components will be categorized based on the complexity and invasiveness of the construction activity similar to ICRAs for airborne pathogens [8]. For instance, modification or repair of plumbing items within one room (e.g., replacing sink fixtures) is less complex than modifications to piping in a wall cavity and/or ceiling space. Additional complexity distinctions are plumbing system components that affect an immediate area of building occupants (e.g., staff locker room and showers) versus changing out a central water heater, storage, or heat exchanging device that impacts a large number of departments and potentially different types of building occupants.

### 3.2. Developing WMC-ICRA Class of Precautions

The Class of Precaution categories are the equivalent of determining the application of control measures within a WMP. A standard ICRA framework for airborne pathogens prescribes a list of ICRMRs [8]. Each ICRMR (i.e., control measure) is organized into four groupings, which subsequently defines each Class of Precaution (I, II, III, or IV) category. Implementation methods (i.e., hazard control options) will be suggested based on water management industry guidance [13,23,24,25], standards [14] and evidence-based practices [2,15,19] that will be subsequently grouped into WMC-ICRA construction and risk mitigation categories.

#### 3.2.1. Monitoring Residual Oxidant (Free or Total)

An effective control for *Legionella* growth and spread in building water systems is disinfectant [13]. The most common disinfectants used by municipal water agencies are chlorine (measured as free residual oxidant, FRO) or monochloramine (measured as total residual oxidant, TRO) [13,19]. A level of FRO between 0.20 ppm and 4.0 ppm or TRO between 0.50 ppm and 4.0 ppm in potable water has been correlated with reducing the risk of growth and spread of disease-causing organisms in building water systems [19]. Digital colorimeters used with the corresponding reagent are the most accurate method of measuring free or total residual oxidant [26].

#### 3.2.2. Monitoring Temperature

Temperature is a significant parameter influencing the growth and spread of waterborne pathogens in building water systems [13]. Simply stated, temperature ranges should keep hot water as hot and cold water as cold [27]. *Legionella* generally grows between 77 °F and 113 °F (25 °C and 45 °C) [13,24]. *Legionella* may grow slowly at temperatures as low as 68 °F (20 °C) [13]. Optimal temperatures for *Legionella* growth are between 85 °F and 108 °F (30 °C and 42 °C) [13]. These ranges are often present in building water systems. Therefore, optimal established water temperatures are cold at ≤77 °F (25 °C) and hot ≥113 °F (45 °C); however, hot water must be below scalding temperatures (see NASEM 2020 Table 4-3 Water Temperature, Risk of Scalding/Burning and *Legionella* Growth Potential) [19] (p. 175) for building occupants. Additionally, long periods of building dormancy create conditions for ambient water temperatures in non-climatized building spaces with temperatures ideal for *Legionella* colonization [15].

#### 3.2.3. Implementing Flushing Protocols

Flushing or purging water through the BWDS is a method used to maintain temperature ranges, reduce water age, and introduce ‘newer’ water with adequate residual oxidant into the system [13]. A flushing protocol includes parameters of minutes of flushing, number of days of the week for flushing, and the number of fixtures to be flushed [13,15,28]. Flushing protocols are highly dependent on the total volume of water contained within the BWDS (i.e., piping size, lengths, water storage, flow rates, and any sediments to be flushed). In the case of construction, a total water volume calculation should be considered for the area under construction to determine the quantity of water necessary to move through the system for 100% system turnover on a periodic basis [13]. For construction projects within existing buildings, weekly flushing was a recommended practice for unoccupied areas, low census areas, construction out of service areas, or room function changes for a minimum 4-min flush for both the hot and cold lines per fixture [28]. For new construction projects ANSI/ASHRAE Standard 188 suggests after disinfection some routine flushing of all fixtures should occur until the building is under normal operating conditions [14].

#### 3.2.4. Utilizing Filtration

Filtration is a means for removing suspended particles from the potable water system before dispensing water at the terminal fixture [13]. Filters, screens, and other devices are commonly applied at the point-of-entry, inline, and/or at point-of use. Filters with a pore size of 0.2 µm or less that comply with industry standardized test methods can provide a barrier to transmission for *Legionella* [13]. The WMP team should carefully review installation and removal of any filtration devices [15,28]. Filter installation will occur after gross purging of the system is undertaken to remove construction soil, sediment, and debris [13].

#### 3.2.5. Installing Physical Barriers

Aerosolization of water from construction activities and from construction equipment with water reservoirs are associated with disease cases [29,30,31]. A physical construction or partition barrier [8] may be necessary to contain and prevent aerosolized water droplets from dispersing into the air, allowing the waterborne pathogen to enter the patient via the inhalation exposure route. When devices are used to spray water, repair fixtures, test fixtures, operationalize misters, or use construction equipment with water reservoirs, any aerosolized water near patient care areas or ventilation air intakes should be contained [13].

#### 3.2.6. Recirculation and Hot Water Storage

Hot water recirculation systems used to maintain hot water temperature and for increasing energy efficiency are a known source of *Legionella* [13]. Building water systems are also designed to store hot water to meet user demand. If these systems are impacted due to the construction project scope, it will be necessary to reduce water age in uncirculated water system components and storage. The volume of water in these components, if impacted, should be considered within the flushing protocols.

#### 3.2.7. Equipment Installation, Cleaning, and Maintenance

Patient and medical equipment devices (e.g., ice machines, cardiac heater-cooler units, or sterile processing equipment) should be scheduled for installation in a timely manner to avoid high water age and premature bacteria growth and spread [13,32,33,34]. Operators must properly clean and maintain all patient and medical equipment using water per the manufacturer’s recommendation prior to use during routine operations [13,32]. Similarly, avoid premature installation of terminal fittings on fixtures (e.g., shower heads and hoses, aerators, faucet flow restrictors, screens, and filters in devices).

#### 3.2.8. Disinfection

Disinfection is a process of killing or inactivating microorganisms and is considered a highly effective method in the control of *Legionella* [13]. The method of disinfection must be reviewed in context of the local municipal water or other source water supplied to the building and the scale and scope of the construction project [14]. Disinfection can be secondary, supplemental, or one time (e.g., hyperchlorination) event [13].

### 3.3. Determining WMC-ICRA Sampling Plans for Verification and Validation Testing

All local WMP team members are responsible for determining the policy and procedures for water testing for construction projects including the number of locations and the types of testing (e.g., chemical or microbial) to be performed [13,24]. ASHRAE 12-2020 defines testing as “conducting a planned sequence of observations or measurements of physical, chemical, or microbial characteristics of water to assess whether conditions throughout the building water system meet the goals set by the group or individual responsible for developing, implementing, and maintaining the water management program” [13] (p. 5). Each project is unique and requires an analysis of the project specific construction scope within the context of any existing or future WMPs for the building to operate to determine verification and validation test methods [14].

## 4. Results

**Figure 1:**
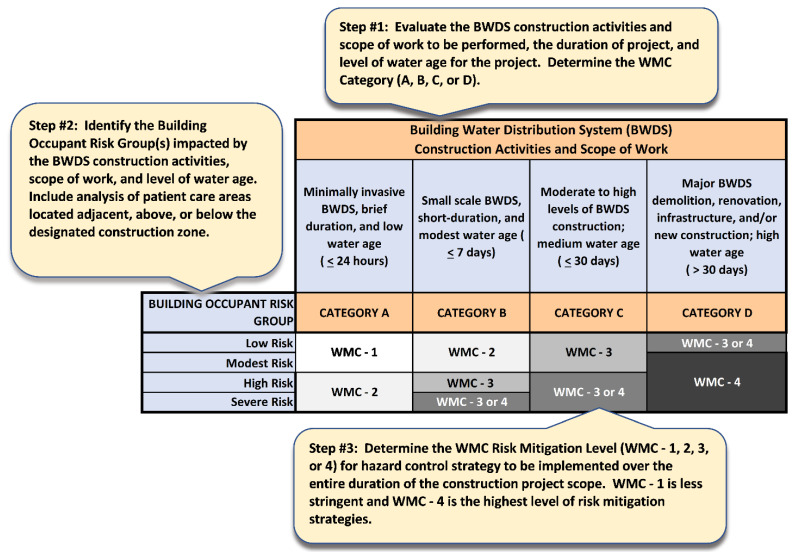
WMC Decision Matrix.

**Figure 2:**
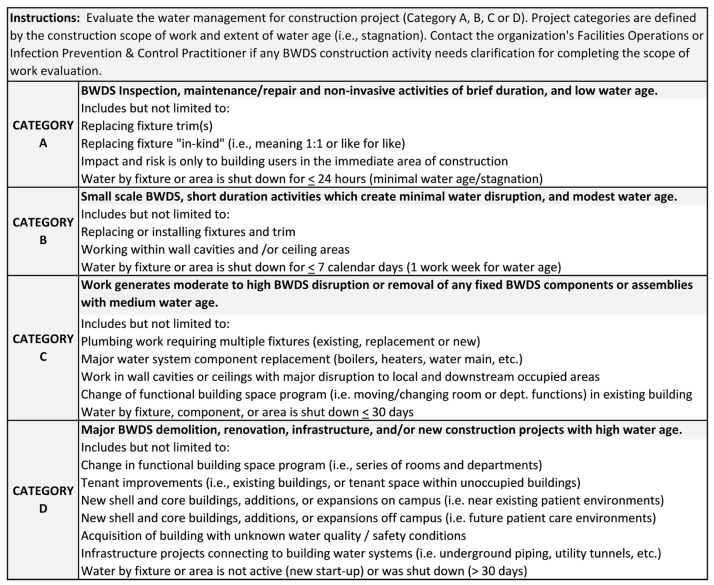
Water Management for Construction ICRA Project Categories for Building Water Distribution Systems (BWDS) [4,7,8,12,13,14,24,25,28,35,36].

**Figure 3:**
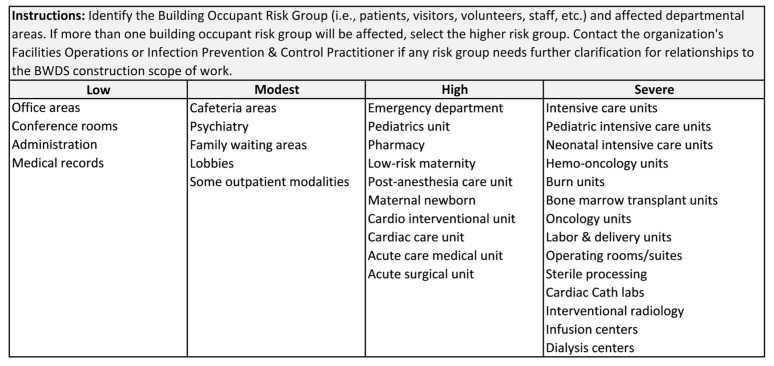
Water Management for Construction ICRA Building Occupant Risk Group Exemplar [4,7,8,36].

**Figure 4:**
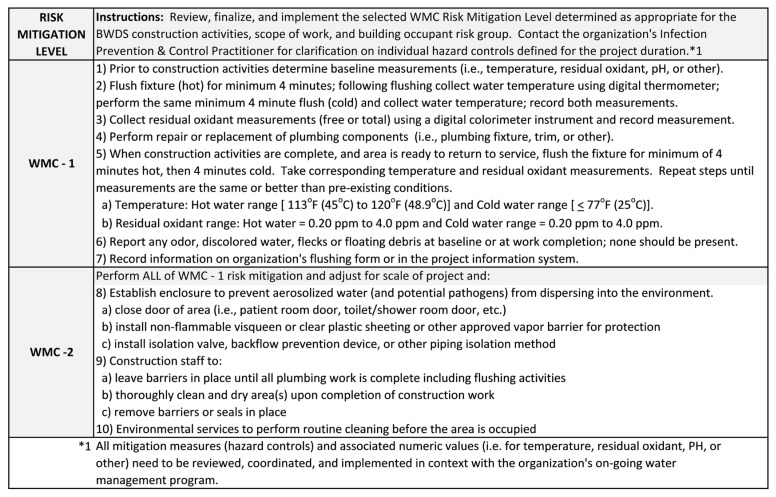
Water Management for Construction Risk Mitigation Levels 1 and 2 [8,13,14,19,24,25,26,28].

**Figure 5:**
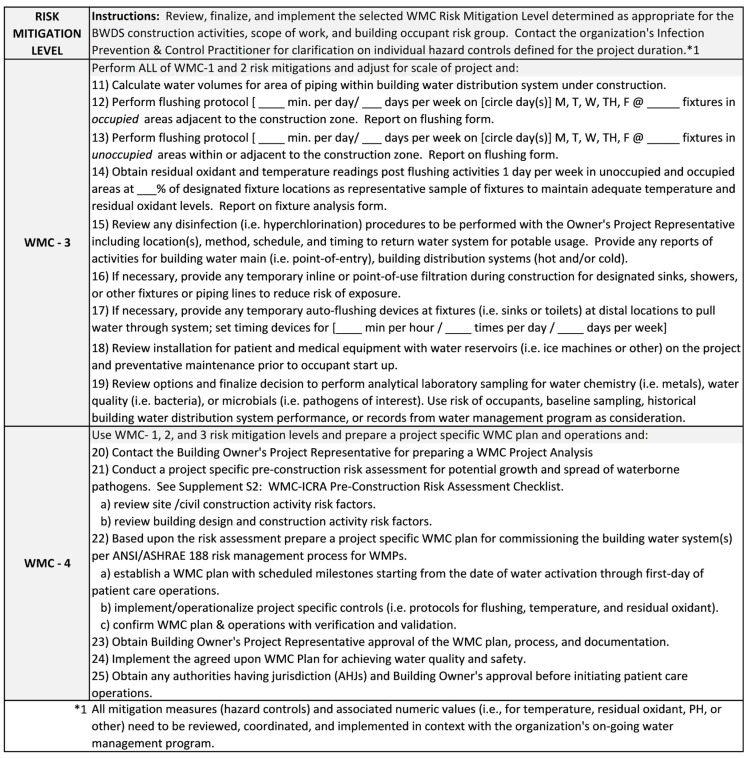
Water Management for Construction Risk Mitigation Levels 3 and 4 [1,8,13,14,19,24,25,28,36].

Considering the methods outlined above, the authors developed an exemplar WMC-ICRA Tool inclusive of Project Category definitions (A, B, C, and D), building occupant risk groups, and WMC Risk Mitigation Levels (WMC-1, 2, 3, and 4) (See Supplemental Document S1: WMC-ICRA Water Quality and Safety Matrix during Construction Activities in Healthcare Settings). Figure 1 depicts a novel WMC Decision Matrix based upon analyzing and selecting: (1) the project category (A, B, C, or D) and scope of BWDS construction to be undertaken; (2) the building occupant risk groups (low, moderate, high, or severe) to be impacted; and (3) the WMC Risk Mitigation Level (WMC-1, 2, 3, or 4) to be implemented. Additionally, a brief description of the intent of each section of the WMC Decision Matrix is outlined below, as well as terms and definitions illustrated in Figure 2, Figure 3, Figure 4 and Figure 5.

### 4.1. WMC-ICRA Project Categories

#### 4.1.1. Construction Scope of Work

To assess the project category (A, B, C, or D) the WMC-ICRA team will identify the scope of work ranging from low construction activity (Category A = inspection, maintenance, and non-invasive activities) to high construction activity (Category D = major demolition, renovation, and new construction projects). Further distinctions may include change of building function (example: renovation of office areas into patient care areas), shell area expansions, or acquisition of a tenant space/building for healthcare operations with unknown history of water quality and safety.

#### 4.1.2. Stratified Water Age Categories

Water age has been found to degrade water quality and safety when water stagnates for 24 h [35], one week (≤7 days) [28], multiple weeks (≤30 days) [14], or after one month (>30 days) [14]. Water age was, therefore, stratified to define dormant water systems within each project type description. Project Category A represents ≤24 h (overnight) low water age projects to be performed with minimal invasive construction activities impacting the water system. Project Category B represents medium water age less than or equal to 7 calendar days representing a one-week construction period (including weekends in which minimal or no construction activities are routinely performed). The one-week period aligns with WMP practices implementing weekly flushing protocols [28]. Project Category C represents high water age ≤ 30 days which aligns with ANSI/ASHRAE Standard 188 Section 8.4.2.1 in which if building occupancy is delayed 2 weeks but less than four weeks (after disinfection), flushing of all fixtures shall again be completed [14]. Project Category D represents highest water age > 30 days which represents significant time for low or no occupancy of existing buildings or start-up of new construction [14,15]. Extensive water age (>30 days) requires a project specific commissioning plan to be developed for implementation. See Figure 2 for WMC-ICRA Project Category definitions.

### 4.2. WMC-ICRA Building Occupant Risk Groups

After determining the project specific BWDS construction scope of work, the infection prevention and control (IPC) practitioner should identify impacted risk groups by building location or department. The building occupant risk groups should be categorized from low to severe depending upon the patient populations served and potential risk of exposure to aerosolized water. Patients who are immunocompromised or receiving specialized diagnostic or treatment for an underlying disease should be considered in the severe risk grouping [10,11,21]. An IPC practitioner can utilize the CDC Water ICRA (WICRA) for Healthcare Settings to assist in evaluation of water sources, modes of transmission, patient susceptibility, and patient exposure [37]. Building occupant risk group designations are evaluated and determined by the local healthcare institution and its WMP Team. See Figure 3 for an exemplar building occupant risk group categories.

### 4.3. WMC Risk Mitigation Levels

The four Risk Mitigation Levels (WMC-1, 2, 3, or 4) contain a checklist of ICRMR methods and procedures to be undertaken throughout the phases of construction until the project is complete. Each WMC Risk Mitigation Level builds mitigation controls from baseline water parameter readings prior to construction to post-construction measurements and testing to return the building water system to normal operating conditions. Figure 4 and Figure 5 summarize the ICRMRs by Risk Mitigation Level WMC-1, 2, 3, and 4.

Larger scale projects (WMC-4 with >30 days of dormancy or new start-up) as indicated in WMC-ICRA Category D should conduct a pre-construction risk assessment (PCRA) (see Supplement S2: WMC-ICRA Pre-Construction Risk Assessment Checklist) for potential growth and spread of waterborne pathogens associated with construction activity risk factors [1]. Following the PCRA, a WMC team would be assembled to create a WMC plan using the ANSI/ASHRAE 188 WMP method [14]. The WMC plan would be operationalized from the date of water activation in the building water system and continue through until the first day of patient care operations. Controls (i.e., flushing protocols, temperature monitoring, and residual oxidant monitoring) would be determined, implemented, and operationalized similar to those listed for WMC-1, 2, and 3, yet scaled for a larger BWDS. The WMC plan and operations would require confirmation through (1) verification that the WMC plan is being implemented as designed, and (2) validation that the WMC plan, when implemented as designed, controls hazardous conditions throughout the building water systems. The building owner’s representative and WMC team would agree upon the WMC plan, operations, and methods of documentation for the project. At the conclusion of the project the WMC plan would be adjusted and transitioned into the on-going WMP and associated team for continued operations. A formal decision would be made by the building owner and any AHJs concerning water quality and safety system approvals before initiating patient care operations.

### 4.4. WMC-ICRA Verification and Validation Testing

Testing events may need to include water quality and safety baseline conditions (e.g., preconstruction testing) as well as testing for same or improved conditions after construction activities (e.g., post construction testing) [25]. Testing may involve water parameters such as temperature or residual oxidant mentioned earlier [13]. Additionally, water chemistry (i.e., metals testing for elevated copper, lead, or other sediments) may be necessary related to older systems or systems with discolored water conditions [15,38]. Further, general bacteria [heterotrophic plate counts (HPC) or total heterotrophic aerobic bacteria (THAB) counts] may be used to determine water quality [27]. Microbial testing should be considered based upon the appropriate AHJ regulatory criteria [23], history of detections within building’s ongoing WMP, epidemiology and clinical surveillance history of the facility, and potential risk to building occupants [25]. It should be noted that post COVID-19 stay-at-home orders when large buildings experienced low and no occupancy, water AHJ shifted policies related to construction, commissioning, and recommissioning [15]. The WMP team implementing a WMC-ICRA should review updated local, state, or federal water policies post COVID-19 for current requirements.

The NASEM 2020 report suggested to reduce the likelihood of aerosol transmission, the absence of *Legionella* should be verified prior to occupancy of the building as part of the commissioning process [19]. If *Legionella* is part of the WMP team’s validation testing criteria to confirm water safety, the CDC Toolkit for Controlling *Legionella* in Common Sources of Exposure [25] states *Legionella* is well controlled in potable water when test results are ≤1 CFU/mL (colony forming unit per milliliter) and cooling tower test results are ≤10 CFU/mL [25]. However, the number of types of chemical or microbial testing as a safety or performance criteria may vary widely with each AHJ [15,20,23,25,27].

## 5. Discussion

Waterborne pathogens from construction activities have been associated with disease cases and deaths since 1965 [1], yet over the past 56 years, the advancement of research, infection prevention practices, hazard recognition and control, and enforcement of standards surrounding transmission of waterborne disease in buildings and construction have not advanced sufficiently to reduce the burden of disease. We created a tool for implementing waterborne pathogen controls for maintenance, repair, and construction projects as a first step to advancing infection control practices in healthcare settings. Our discussion describes the alignment of the WMC-ICRA tool and impacts on regulatory requirements, non-regulatory guidance and best practices, infection prevention, and industrial hygiene hazard controls, for establishing a safe BWDS from construction activities to protect the health and safety of building occupants.

### 5.1. Health and Built Environment Perspective

The innovation of the sanitary water supply and sewage system increased the standards for water quality and safety [33]. The Safe Drinking Water Act of 1974 codified the right of every person in the US to have safe potable water delivered to the building perimeter for any residential or public building, including healthcare facilities [39]. Although related, drinking water quality focuses on the human exposure route of ingestion, not inhalation. Water safety can sometimes be distinguished from water quality as it pertains to the inhalation route of exposure, via aerosolized water sources in the environment [33]. Water quality and safety in a healthcare environment for treating patients is essential for protecting the health, safety and welfare of the patient while receiving care [20]. To reduce the risk of patient exposure, healthcare WMP teams must manage building water quality and safety during all maintenance, repair, and construction activities [5,14]. Similar safety precautions are in place for airborne pathogens and the same must be achieved for waterborne pathogen exposure [21].

Water management is the accepted best practice to reduce *Legionella* growth and spread in large building water systems inclusive of healthcare operations [14,20]. Maintaining a WMP is now a requirement for any acute care, skilled nursing, or critical care access hospital seeking US Federal reimbursement for patient care operations [20]. The Center for Medicare and Medicaid Services (CMS) issued a Survey and Certification (S&C) Group Memorandum (CMS S&C Memo 17–30) Requirement to Reduce *Legionella* Risk in Healthcare Facility Water Systems to Prevent Cases and Outbreaks of Legionnaires’ Disease, for use in healthcare settings with overnight stay (≥24 h) [20]. The CMS S&C Memo 17–30 [20] identifies *Legionella* and other opportunistic waterborne pathogens (e.g., *Pseudomonas*, *Acinetobacter*, *Burkholderia*, *Stenotrophomonas*, *Nontuberculous mycobacteria*, and *fungi*). The healthcare provider is instructed to utilize ANSI/ASHRAE Standard 188 which in turn directly states the building owner must survey and develop risk management for construction project types (i.e., renovation, additions, modifications to existing buildings, and new construction) [14]. The FGI Guidelines has required the implementation of a construction ICRA to address air and waterborne pathogens since 2014 for all renovations and new construction projects [5]. These AHJs indicate healthcare providers must have a policy and plan of action for risk assessment and control of waterborne pathogens around construction activities. The WMC-ICRA tool provided here gives healthcare organizations a valuable template that will help local water management team members develop a uniform policy and process for ease of implementation.

After ICRAs for airborne pathogens were introduced into the construction process, the construction materials industry responded with innovative control techniques for dust particles [8]. Today general contractors and air ventilation subcontractors routinely purchase products for dust control (e.g., sticky mats, mobile air machines with filtration, dehumidifiers, flood kits, temporary wall barriers, environmental containment units, and containment tape and sealants, among numerous other products) [6,40]. These products were invented for the primary purpose of protecting patients from exposure to airborne particulate matter. Similar materials (i.e., fire retardant polyurethane) and assemblies [8] may be appropriate for adaptation for containment of aerosolized building water. With the implementation of WMC-ICRA, designers, contractors, IPC practitioners and facility management teams should use known control methods for waterborne pathogens, as identified throughout this manuscript. However, these teams should anticipate the introduction of new devices as well as new alternative and novel test methods [25] for implementing water hazard controls during construction activities. These future devices and test methods are not yet identified, less defined, or manufactured and will need to be robustly reviewed for field application.

### 5.2. Infection Prevention and Control Perspective

#### 5.2.1. Increased Planning and Consistent Implementation

Risk assessment allows for increased planning, systematic analysis, establishment of hazard controls, and allows for consistent implementation to prevent waterborne pathogen transmission. Implementing the WMC-ICRA tool will likely decrease the incidence and the burden of disease outbreaks associated with waterborne pathogens and construction activities. Waterborne pathogens such as *Legionella,* Nontuberculous mycobacteria (NTM), *Stenotrophomonas maltophilia*, and *Acinetobacter baumannii* are not simply water contaminants, but colonizers of water [41]. These pathogens survive and thrive in natural and potable water. Case studies demonstrate these colonizers of water during and after construction activities are pathogenic and virulent, particularly with immunocompromised patients [1]. Incidence of disease from NTM and other opportunistic pathogens found in water and soil, appears to be increasing. Baker et al. [42] reported an outbreak at a newly constructed hospital. The outbreak was linked to a colonized water supply and was identified using cultures of biofilms from hospital water outlets which grew *Mycobacterium abscessus* complex (MBAC). Astute clinicians thought to check an environmental source, building water, as part of their outbreak investigation. Outbreaks associated with common water exposures are difficult to investigate, and frequently overlooked [11].

Epidemiologic and environmental surveillance for waterborne disease associated with healthcare construction activities is challenging, which obscures our ability to recognize, evaluate and control hazards. Water within the infection preventionist’s index of suspicion in a newly opened building may seem low, yet when these events occurred in a healthcare setting, they resulted in a high number of disease cases (mean of *n* = 26.5) and deaths (mean of *n* = 4.3) [1]. The mean duration of an outbreak in a healthcare building after undergoing construction activities is 17.3 months [1]. This magnitude of disease transmission would be considered a large outbreak event in any healthcare institution. The WMC-ICRA can help the team to anticipate where risk of disease transmission, morbidity and mortality, may occur such as departments serving immunocompromised patients [28].

Additionally, WMC-ICRA should allow for proper building water systems management from the date of water activation during construction by early adoption of the WMP already required by CMS and TJC, among others [15,20,43]. Waterborne pathogens proliferate in biofilms, which propagate during water stagnation [13]. Improper building water distribution design and commissioning can lead to increased colonization of bacteria such as *Legionella* [1,13,19]. The effectiveness of temperature and disinfectant as risk mitigation strategies diminishes over time without adequate water movement [44]. During construction, the BWDS is filled with water (i.e., water activation) and may be left for weeks or months without use before the building is occupied with employees and patients [13]. One mitigation measure may involve the delay of water activation in building distribution piping to reduce water age and stagnation [36]. Furthermore, the WMC-ICRA tool will assist an IPC practitioner to recommend flushing activities, temperature and disinfection checks, validation testing, and validation responses to improve water quality and safety and prevent potential outbreaks. 

The IPC practitioner will be an important WMP team member [14,33] to clarify the need for chemical or microbial testing. As stated previously, the WMP team will determine the policy and procedures for water testing for construction projects, which includes the type of tests and pathogens of interest. Appropriate pathogens of interest and suitable validation testing requirements can vary widely from country, state, province, and municipality [15,20,23]. The IPC practitioner can utilize history of clinical disease at the facility and the microbial testing within a WMP for on-going operations as a starting point. For example, in a US healthcare facility, if *Legionella* is the pathogen of interest within the organization’s WMP, the IPC practitioners would familiarize themselves with the CDC’s routine testing for *Legionella* [25] used outside of the context of an outbreak investigation. The CDC described a multifactorial approach for interpreting water safety performance criteria [25] (Figure 1, p. F5). The multifactorial approach consists of (a) interpreting CFU/mL concentrations to describe *Legionella* growth and spread as uncontrolled, poorly controlled, and well controlled; (b) assessing detections as originating from multiple locations (e.g., fixture locations) or common sources (e.g., water heaters); and (c) the number of rounds of testing without *Legionella* detections. Standardization of test methods for other microbial hazards in the context of water management are forthcoming in ASHRAE Standard 514 Risk Management for Building Water Systems: Physical Chemical and Microbial Hazards currently in the public review comment phase [18].

Further, WMC-ICRA can also provide guidance to safely recommission building water systems following catastrophic events. During lockdown strategies to combat the SARS-CoV-2 pandemic, multiple buildings, including offices, healthcare clinics, and universities, were closed for months before reopening for public occupancy [15]. If the BWDS and water remained unused during this time the building was recommended to undergo a re-commissioning event to restore water quality and safety prior to occupancy [15]. The CDC, the Environmental Protection Agency (EPA), and other AHJs prepared guidance documents related to reopening buildings safely after prolonged shutdown for low or no use of the BWDS [15]. Physical, chemical, or microbial hazards such as *Legionella*, NTM, disinfection by-products, lead and copper leaching, and sewer gases, among others, can emerge following building water system shutdowns [15]. Risk assessment and the timely application of hazard controls prior to re-occupancy should reduce risk of emerging disease cases and outbreaks from occurring under such conditions [15].

A WMP complemented with the use of WMC-ICRA has the potential to prevent the transmission of the disease agent before the water can be a vector to the susceptible person. The strength of this tool is its potential to positively influence health outcomes. While viruses, such as SARS-CoV-2, are novel, bacterial co-infections with viral illnesses are not [45]. The need for vigilance to prevent transmission of *Legionella* and other waterborne pathogens in a healthcare setting where viral illness may be rampant, (i.e., during the ongoing SARS-CoV-2 pandemic) is paramount. Recent case studies are beginning to examine co-infections of *Legionella* and SARS-CoV-2, particularly among patients with multiple co-morbidities [46,47]. Allam et. al. [47] reported three of the seven cases of co-infection during their monthlong surveillance were healthcare associated. All seven patients co-infected with SARS-CoV-2 and *Legionella* required intensive care unit admission and had a high case-fatality rate. These outbreaks highlight the need to continue infection prevention surveillance and mitigation activities to prevent the colonization of *Legionella*. Importantly, influenza circulates annually and leads to thousands of hospitalizations. Bacterial co-infections with this virus are numerous and deadly [48].

#### 5.2.2. Extending Patient Safety to Other WMP Team Members

The WMC-ICRA functions as a quality and safety tool for multidisciplinary teams to quantify risk and perform hazard control. The WMC team can then use data collected from the tool to inform decision making and balance mitigation efforts with project constraints. Each healthcare departmental service line (e.g., administration, clinical nursing, engineering, facility management, and central sterilization, among others) has responsibilities to keep the WMP both comprehensive and effective [14]. As the Agency for Healthcare Research and Quality states, “Unfortunately, design-related vulnerabilities that adversely impact patient safety are sometimes inadvertently built into the physical environment during the planning, design, and construction of health care facilities. These problems are difficult and expensive to address once a facility has been built and occupied” [49]. The WMC-ICRA tool was designed and created to combat this very vulnerability for water contaminant transmission.

Integration of the WMC-ICRA tool will increase IPC communication and exchange of information to a wide variety of internal and external stakeholder disciplines, even those without a deep background in healthcare or disease prevention, to participate effectively and efficiently throughout the construction project. Moving water management practices beyond facility operations and toward design and construction increases opportunity for engagement with the building owner’s construction management team including the general contractor and subtrades [1,19]. The WMC team can be prescribed roles and responsibilities to optimize water quality, safety, and efficiency during building commissioning, rather than inheriting an unsafe BWDS for patient care operations. Building upon the technical framework of ICRAs for airborne pathogens [8] avoids introducing a new or foreign process to the construction industry. Using the WMC-ICRA tool will engage the general contractor and subtrades to actively monitor water distribution control points, understand flushing protocols, and measure temperature and residual oxidant to assure the BWDS can reduce the risk of contributing to premature waterborne pathogen growth and spread. Their decisions and input will help optimize the building for water quality and safety. Over time this will increase the design and construction industry’s knowledge for developing a project specific water activation schedule including water main activation, fixture installation, flushing protocols, disinfection procedures, and the coordination of these activities around existing and future building occupants.

#### 5.2.3. Increasing IPC Practitioner Core Competencies

The APIC Competency Model [50] has many knowledge areas in alignment with the application of the WMC-ICRA tool. The WMC-ICRA advances the IPC practitioner’s core competencies and domains in the areas of leadership, risk assessment and risk reduction, epidemiology and surveillance, and the evaluation of research. A WMP team is a multidisciplinary, systems-based approach to managing risk [14,28]. The IPC practitioner must collaborate with other leaders and share common goals of patient safety, infection prevention, fiscal responsibility, and innovation [50]. The IPC practitioner has a unique skill set to lead the WMC team in education, analysis, and interventions which ultimately benefits patient safety and impacts building asset management [50]. Construction and water management are areas considered within the IPC expert category of competency. The WMC-ICRA offers a user-friendly and reliable tool to apply to projects to progress IPC skills toward expert status. Billings et al. [50] mentions water management, risk assessment, *Legionella* mitigation efforts, and knowledge of FGI Guidelines as topics within the wide spectrum of knowledge, skills, and abilities critically important to building IPCs as institutional leaders and subject matter experts who can see the bigger picture and positively impact patient safety.

### 5.3. Industrial Hygiene Perspective

WMC-ICRA promotes responsible water management from the moment water is activated within the BWDS and before it comes in contact with building occupants. Test and inspection of building engineering systems (i.e., structural, mechanical, electrical, and medical gas piping, among others) are commonly performed and required per building codes prior to occupancy to assure safe patient care operations [5,51]. Previous guidance for response to *Legionella* or waterborne pathogens outbreaks suggests decision making to test water should occur after a disease case has been identified [24]. The current authors argue that best practices would instead highlight the importance of anticipation and recognition of the waterborne disease hazards in healthcare settings. Since water is a known source for disease in association with construction activities, a prevention strategy should be implemented [11]. The WMC-ICRA provides a framework to protect building occupants from these known threats, prior to occupancy. The tool is not intended to aid in the response to an outbreak or a disease case that has occurred during on-going building operations. Instead, the WMC-ICRA tool is intended to afford the water management team the opportunity to define, perform, verify, and validate a water management program inclusive of construction activities based on previous findings [1] demonstrating the need to protect all current or future building occupants (i.e., construction workers, clinical staff, administrative and technical staff, visitors, and patients). This would not only meet the requirements of CMS, TJC, and other AHJ, but would meet the intent of the Occupational Safety and Health Act of 1970 [11,52]. While there is no specific standard requiring prevention of *Legionella* exposure in the workplace, the General Duty Clause requires employers to provide a work environment “free from recognized hazards that are causing or likely to cause death or serious physical harm” including *Legionella* [53].

Employers are strongly encouraged to familiarize themselves with guidance from the Occupational Safety and Health Administration on the prevention of *Legionella* exposure and associated disease, which focuses on the maintenance, cleaning, and disinfection of water systems, as would typically be managed through a WMP in healthcare settings [52]. Though healthcare WMPs and the WMC-ICRA were created primarily to prevent negative outcomes in patients, healthcare and construction workers are also potentially exposed and vulnerable to *Legionella* and other pathogens, and will benefit from the anticipation, recognition, evaluation, and control of these hazards in the workplace. Industrial Hygienists are trained to focus on engineering and work-practice controls aimed at preventing exposure before any infection is detected, practices that offer “the best possibility for prevention and control of Legionnaires’ disease” [54]. The WMC-ICRA aids the practicing industrial hygienist in these prevention efforts by providing a systematic framework for the analysis of water systems during construction activities, when the system is more vulnerable, and conditions are more ideal for pathogen propagation [13,44]. Although healthcare workers and construction workers have a smaller documented burden of *Legionella* disease than patients [1], occupational exposures and disease cases are described in the literature [30,32,55] and disease may be underreported among workers compared to patients in healthcare settings [52].

### 5.4. Limitations

The WMC-ICRA tool, tables, and definitions presented by the authors are only an exemplar and their application requires each organization to review the contents for appropriateness and application in conjunction with the organization’s water management program and water management for construction practices. Every building water system has unique attributes which can create different results and must be evaluated by the responsible water management program team acting on behalf of the building owner and its population risk groups. Modifications will be necessary for local, state, or federal regulatory requirements including microbial test methods, as well as any numeric values (e.g., temperature, residual oxidant, etc.) within the organization’s on-going building water management program. The user and their organization assume the sole risk and full responsibility for implementation of such practices and consequences of implementation in healthcare environments. The authors make no representations or warranties about the suitability, completeness, reliability, legality, accuracy, or appropriateness of the information provided to reduce the likelihood of waterborne pathogens present in building water systems, the disease cases, injury, or deaths that may emerge from such building water systems. Another limitation is the lack of water management for construction training. As a WMC-ICRA strategy emerges there will be knowledge gaps to address, as well as an opportunity for healthcare industry improvements in the area of water safety.

## 6. Conclusions

Protection of patients, visitors, and staff from a potentially unsafe BWDS is essential during renovation and new construction inclusive of any adjacent departments to a renovation area and surrounding occupied buildings on the construction site [1,5,14]. The CDC reported 35% of issues concerning water safety in buildings comes from sources outside of the building, citing compromises to the municipal water system in the form of water main brakes and construction [2]. A *Legionella* risk management plan is to be established and modified throughout the project from early planning, during each phase of design and construction, and during commissioning [5,14]; however, no tool has been developed to assist the approximately 6000 hospitals and 14,000 skilled nursing facilities in the US to perform a waterborne pathogen pre-construction and infection control risk assessment. Each healthcare institution implementing a WMP is required to include provisions for maintenance, repair, and construction activities [14]. Our recommendation is for each healthcare organization to review and develop a policy leveraging the methods demonstrated within the WMC-ICRA tool and customize their efforts to the site specific/organizational WMP to address five potential gaps:

1. Reduce the likelihood of a disease case, injury, or death from a healthcare associated infection emerging from exposure to the BWDS during and after construction activities;
2. Reduce the likelihood of waterborne pathogen growth and spread in a BWDS undergoing any form or range of project involving construction activities;
3. Improve regulatory alignment with healthcare standards and policies requiring risk mitigation for waterborne pathogen growth and spread during construction activities;
4. Expand use of existing ICRA framework commonly used for airborne pathogens to align with waterborne pathogens for ease of healthcare industry implementation;
5. Extend collaboration of water management programs and teams to allied industries from planning, design, and construction to include water safety as a common protection for building occupants as part of licensed professionals’ duty and standard of care to protect the health, safety, and welfare of the public related to any renovated or newly constructed BWDS and its components.

## Data Availability

Not applicable.

## Supplementary Materials

The following supporting information can be downloaded at: https://www.mdpi.com/article/10.3390/idr14030039/s1, Supplemental File S1: WMC-ICRA Water Quality and Safety Matrix for Construction Activities in Healthcare Settings; Supplemental File S2: WMC-ICRA Pre-Construction Risk Assessment Checklist.
