# Supplementary material for: Reducing the Risk of Healthcare Associated Infections from Legionella and Other Waterborne Pathogens Using a Water Management for Construction (WMC) Infection Control Risk Assessment (ICRA) Tool
Source: Infect Dis Rep. 2022 May 6;14(3):341–59. doi: 10.3390/idr14030039 (PMC9149880; doi:10.3390/idr14030039)
Supplement: Supplementary file 1 [file idr-14-00039-s001.zip › WMC-ICRA Supplement S1 Rev2.pdf]

SUPPLEMENT S1

WATER MANAGEMENT FOR CONSTRUCTION (WMC) - INFECTION CONTROL RISK ASSESSMENT (ICRA)

Building Water Distribution System (BWDS)

Water Quality and Safety Matrix for Construction Activities in Healthcare Settings

Exemplar defining Construction Project Categories, Building Occupant Risk Groups, and WMC Risk Mitigation Levels for Construction Activities

Disclaimer: This is an example WMC-ICRA document only and requires each organization to review its contents for appropriateness and application in conjunction with the organization's water management program and water management for construction practices. The user / organization assumes the sole risk and full responsibility for implementation of such practices and consequences of implementation in healthcare environments. The authors make no representations or warranties about the suitability, completeness, reliability, legality, accuracy, or appropriateness of the information provided to reduce the likelihood of waterborne pathogens present in building water distribtuion systems (BWDS), the disease cases, or deaths that may emerge from such building water systems. Every building water system has unique attributes that can create different results and must be evaluated by the responsible water management program team acting on behalf of the building owner. Modifications will be necessary for local, state, or federal regulatory requirements, as well as any numeric values (ex. temperature, residual oxidant, etc.) within organization's building water management program.

TABLE S1 **Instructions:** Evaluate the water management for construction project (Category A, B, C or D). Project categories are defined by the construction scope of work and extent of water age (i.e., stagnation). Contact the organization's Facilities Operations or Infection Prevention & Control Practitioner if any BWDS construction activity needs clarification for completing the scope of work evaluation.

**CATEGORY A**	**BWDS Inspection, maintenance/repair and non-invasive activities of brief duration, and low water age.**<br>Includes but not limited to:<br>Replacing fixture trim(s)<br>Replacing fixture "in-kind" (i.e. meaning 1:1 or like for like)<br>Impact and risk is only to building users in the immediate area of construction<br>Water by fixture or area is shut down for ≤ 24 hours (minimal water age/stagnation)
**CATEGORY B**	**Small scale BWDS, short duration activities which create minimal water disruption, and modest water age.**<br>Includes but not limited to:<br>Replacing or installing fixtures and trim<br>Working within wall cavities and /or ceiling areas<br>Water by fixture or area is shut down for ≤ 7 calendar days (1 work week for water age)
**CATEGORY C**	**Work generates moderate to high BWDS disruption or removal of any fixed BWDS components or assemblies with medium water age.**<br>Includes but not limited to:<br>Plumbing work requiring multiple fixtures (existing, replacement or new)<br>Major water system component replacement (boilers, heaters, water main, etc.)<br>Work in wall cavities or ceilings with major disruption to local and downstream occupied areas<br>Change of functional building space program (i.e. moving/changing room or dept. functions) in existing building<br>Water by fixture, component, or area is shut down ≤ 30 days
**CATEGORY D**	**Major BWDS demolition, renovation, infrastructure, and/or new construction projects with high water age.**<br>Includes but not limited to:<br>Change in functional building space program (i.e. series of rooms and departments)<br>Tenant improvements (i.e. existing buildings, or tenant space within unoccupied buildings)<br>New shell and core buildings, additions, or expansions on campus (i.e. near existing patient environments)<br>New shell and core buildings, additions, or expansions off campus (i.e. future patient care environments)<br>Acquisition of building with unknown water quality / safety conditions<br>Infrastructure projects connecting to building water systems (i.e. underground piping, utility tunnels, etc.)<br>Water by fixture or area is not active (new start-up) or was shut down (> 30 days)

TABLE S2 **Instructions:** Identify the Building Occupant Risk Group (i.e., patients, visitors, volunteers, staff, etc.) and affected departmental areas. If more than one building occupant risk group will be affected, select the higher risk group. Contact the organization's Facilities Operations or Infection Prevention & Control Practitioner if any risk group needs further clarification for relationships to the BWDS construction scope of work.

EXAMPLE ONLY: Building Occupant Risk Groups must be evaluated and classified by each organization prior to risk evalation.

Low	Modest	High	Severe
Office areas	Cafeteria areas	Emergency department	Intensive care units
Conference rooms	Psychiatry	Pediatrics unit	Pediatric intensive care units
Administration	Family waiting areas	Pharmacy	Neonatal intensive care units
Medical records	Lobbies	High-risk maternity	Hemo-oncology units
	Some outpatient modalities	Maternal newborn	Burn units
		Post-anesthesia care unit	Bone marrow transplant units
		Cardio interventional unit	Oncology units
		Cardiac care unit	Labor & delivery units
		Acute care medical unit	Operating rooms/suites
		Acute surgical unit	Sterile processing
			Cardiac Cath labs
			Interventional radiology
			Infusion centers
			Dialysis centers

SUPPLEMENT S1

WATER MANAGEMENT FOR CONSTRUCTION (WMC) - INFECTION CONTROL RISK ASSESSMENT (ICRA)

Building Water Distribution System (BWDS)

Water Quality and Safety Matrix for Construction Activities in Healthcare Settings

Exemplar defining Construction Project Categories, Building Occupant Risk Groups, and WMC Risk Mitigation Levels for Construction Activities

TABLE S3 Instructions: Use the WMC Decision Matrix to determine appropriate WMC Risk Mitigation Level. After evaluationg the WMC Category (A B, C, or D) and the Building Occupant Risk Group (low, modest, high, or severe), determine the appropriate WMC Risk Mitigation Level (WMC - 1, 2, 3, or 4). See Table 4 for WMC Risk Mitigation Level descriptions.

<table>
<tr><th></th><th colspan="4">Water Management for Construction (WMC) Decision Martrix
Building Water Distribtuion System (BWDS) Construction Activities and Scope of Work</th></tr>
<tr><td></td><td>Minimally invasive BWDS, brief duration, and low water age (≤ 24 hours)</td><td>Small scale BWDS, short-duration, and modest water age (≤ 7 days)</td><td>Moderate to high levels of BWDS construction, and medium water age (≤ 30 days)</td><td>Major BWDS demolition, renovation, infrastructure, and/or new construction with high water age (> 30 days)</td></tr>
<tr><td>BUILDING OCCUPANT RISK GROUP</td><td>CATEGORY A</td><td>CATEGORY B</td><td>CATEGORY C</td><td>CATEGORY D</td></tr>
<tr><td>Low Risk</td><td rowspan="2">WMC - 1</td><td rowspan="2">WMC - 2</td><td rowspan="2">WMC - 3</td><td>WMC - 3 or 4</td></tr>
<tr><td>Modest Risk</td><td rowspan="3">WMC - 4</td></tr>
<tr><td>High Risk</td><td rowspan="2">WMC - 2</td><td>WMC - 3</td><td rowspan="2">WMC - 3 or 4</td></tr>
<tr><td>Severe Risk</td><td>WMC - 3 or 4</td></tr>
</table>

TABLE S4 **Instructions:** Review, finalize, and implement the selected WMC Risk Mitigation Level determined as appropriate for the BWDS construction activities, scope of work, and building occupant risk group. Contact the organization's Infection Prevention & Control Practitioner for clarification on individual hazard controls defined for the project duration.

WMC RISK MITIGATION LEVELS*1	
WMC RISK MITIGATION LEVEL	**Instructions:** Identify WMC Risk Mitigation Levels below; each risk mitigation level builds upon the previous for an inclusive list of WMC hazard controls.
WMC - 1	1) Prior to construction activities determine baseline measurements (i.e. temperature, residual oxidant, pH, or other).
	2) Flush fixture (hot) for minimum 4 minutes; following flushing collect water temperature using digital thermometer; perform the same minimum 4 minute flush (cold) and collect water temperature; record both measurements.
	3) Collect residual oxidant measurements (free or total) using a digital colorimeter instrument and record measurement.
	4) Perform repair or replacement of plumbing components (i.e. plumbing fixture, trim, or other).
	5) When construction activities are complete, and area is ready to return to service, flush the fixture for minimum of 4 minutes hot, then 4 minutes cold. Take corresponding temperature and residual oxidant measurements. Repeat steps until measurements are the same or better than pre-existing conditions.
	a) Temperature: Hot water range [113°F (45°C) to 120°F (48.9°C)] and Cold water range [≤ 77°F (25°C)].
	b) Residual oxidant range: Hot water = 0.20 ppm to 4.0 ppm and Cold water range = 0.20 ppm to 4.0 ppm.
	6) Report any odor, discolored water, flecks or floating debris at baseline or at work competion; none should be present.
	7) Record information on organization's flushing form or in the project information system.
WMC - 2	Perform ALL of WMC - 1 risk mitigation and adjust for scale of project and:
	8) Establish enclosure to prevent aerosolized water (and potential pathogens) from dispersing into the environment.
	a) close door of area (i.e. patient room door, toilet/shower room door, etc.)
	b) install non-flammable visqueen or clear plastic sheeting or other approved vapor barrier for protection
	c) install isolation valve, backflow prevention device, or other piping isolation method
	9) Construction staff to:
	a) leave barriers in place until all plumbing work is complete including flushing activities
	b) thoroughly clean and dry area(s) upon completion of construction work
	c) remove barriers or seals in place
	10) Environmental services to perform routine cleaning before the area is occupied.

TABLE S4 (continued)

**WMC - 3**	Perform ALL of WMC - 1 and 2 risk mitigation levels and adjust for scale of project and:<br>11) Calculate water volumes for area of piping within building water distribution system under construction.<br>12) Perform flushing protocol [____ min. per day/ ___ days per week on [circle day(s)] M, T, W, TH, F @ _____ fixtures in *occupied* areas adjacent to the construction zone. Report on flushing form.<br>13) Perform flushing protocol [____ min. per day/ ___ days per week on [circle day(s)] M, T, W, TH, F @ _____ fixtures in *unoccupied* areas within or adjacent to the construction zone. Report on flushing form.<br>14) Obtain residual oxidant and temperature readings post flushing activities 1 day per week in unoccupied and occupied areas at ___% of designated fixture locations as representative sample of fixtures to maintain adequate temperature and residual oxidant levels. Report on fixture analysis form.<br>15) Review any disinfection (i.e. hyperchlorination) procedures to be performed with the Owner's Project Representative including location(s), method, schedule, and timing to return water system for potable usage. Provide any reports of activities for building water main (i.e. point-of-entry), building distribution systems (hot and/or cold).<br>16) If necessary, provide any temporary inline or point-of-use filtration during construction for designated sinks, showers, or other fixtures or piping lines to reduce risk of exposure.<br>17) If necessary, provide any temporary auto-flushing devices at fixtures (i.e. sinks or toilets) at distal locations to pull water through system; set timing devices for [____ min per hour / ____ times per day / ____ days per week]<br>18) Review installation for patient and medical equipment with water reservoirs (i.e. ice machines or other) on the project and preventative maintenance prior to occupant start up.<br>19) Review options and finalize decision to perform analytical laboratory sampling for water chemistry (i.e. metals), water quality (i.e. bacteria), or microbials (i.e. pathogens of interest). Use risk of occupants, baseline sampling, historical building water distribution system performance, or records from water management program as consideration.
**WMC- 4**	Use WMC- 1, 2, and 3 risk mitigation levels and prepare a project specific WMC plan and operations and:<br>20) Contact the Building Owner's Project Representative for preparing a WMC Project Analysis<br>21) Conduct a project specific pre-construction risk assessment for potential growth and spread of waterborne pathogens.<br>See Supplement S2: WMC-ICRA Pre-Construction Risk Assessment Checklist.<br>a) review site /civil construction activity risk factors.<br>b) review building design and construction activity risk factors.<br>22) Based upon the risk assessment prepare a project specific WMC plan for commissioning the building water system(s) per ANSI/ASHRAE 188 risk management process for WMPs.<br>a) establish a WMC plan with scheduled milestones starting from the date of water activation through first-day of patient care operations.<br>b) implement/operationalize project specific controls (i.e., protocols for flushing, temperature, and residual oxidant).<br>c) confirm WMC plan & operations with verification and validation.<br>23) Obtain Building Owner's Project Representative approval of the WMC plan, process, and documentation.<br>24) Implement the agreed upon WMC Plan for achieving water quality and safety.<br>25) Obtain any authorities having jurisdiction (AHJs) and Building Owner's approval before initiating patient care operations.

Footnotes:

*1 All mitigation measures (hazard controls) and associated numeric values (i.e., temperature, residual oxidant, pH, or other) need to be reviewed, coordinated and implemented in context with the organization's on-going water management program.

Article Citation: Scanlon MM, Gordon JL, Tonozzi AA, and Griffin SC (2022). Reducing the Risk of Healthcare Associated Infections from *Legionella* and Other Waterborne Pathogens Using a Water Management for Construction (WMC) Infection Control Risk Assessment (ICRA) Tool. *Infectious Disease Reports*, 14 (3). The following references from the article were used to inform the WMC Decision Matrix and Tables [1,4,6,7,8,9,12,13,14,19,24,25,26,28,35,36,44].
