# Supplementary material for: Reducing the Risk of Healthcare Associated Infections from Legionella and Other Waterborne Pathogens Using a Water Management for Construction (WMC) Infection Control Risk Assessment (ICRA) Tool
Source: Infect Dis Rep. 2022 May 6;14(3):341–59. doi: 10.3390/idr14030039 (PMC9149880; doi:10.3390/idr14030039)
Supplement: Supplementary file 1 [file idr-14-00039-s001.zip › WMC-ICRA Supplement S2 Rev2.pdf]

SUPPLEMENT S2: WMC-ICRA PRE-CONSTRUCTION RISK ASSESSMENT CHECKLIST (page 1 of 2)

Project Number:	Facility:
Project Name:	Department/Location:
Date:	Project Scope of Work Description:

Use this document in conjunction with Water Management for Construction (WMC) Infection Control Risk Assessment (ICRA) tool for improving water quality and safety associated with construction activities. The objective is to reduce risk of waterborne pathogen growth and spread in building water distribution systems from construction activities (i.e., any new building, or any renovation, alteration, and demolition). Please identify all risk factors within the construction project scope. The WMC PRCA checklist should be reviewed by an infection prevention and control practitioner and facility construction project manager to determine WMC-ICRA Construction Project Category, Building Occupant Risk Groups, and Risk Mitigation Level. Describe additional information in the Note(s) Section to clarify extent of construction project scope.

YES	NO	SITE WMC RISK FACTORS	NOTES SECTION
		**Excavation**	
		Potential for soil and sediment invasion	
		Describe location(s)	
		**Underground utility connections**	
		Potential for soil and sediment invasion	
		**Repressurization of building main / point-of-entry water system**	
		Will the building main entry be shut-down or experience re-pressurization?	
		**Site/Civil Water service disruption**	
		New construction tie-ins	
		Replacement valves	
		Hydrants	
		Meters	
		Pumping failures	
		Pipeline breaks	
		Other system repairs	
		Emergency conditions	
		**Lengthy underground piping connections**	
		Site routing of water utility piping	
		Fire hydrant locations and piping routing with dead-ends	
		Distance from building main connection to the street connection/invert	
		**Vibration activities**	
		Pile Driving/structural foundation	
		Jackhammering	
		Saw cutting	
		What buildings are these activities adjacent to?	
		**Demolition Activities**	
		Creates air plumes of dust or water aerosoles toward patient care areas	
		Drift of debris toward cooling towers	
		Drift of debris toward HVAC intake vents	
		**Demolishing building water system components**	
		Impacting other building water supply connection points	
		Other Site/Civil utiity demolition activities	
		**Demolishing underground tunnels**	
		Utility	
		Walking/ passage / transportation	
		**Construction equip with water reservoirs (i.e,. typically spray activities)**	
		Water tankers	
		Paving equipment	
		Spray nozzles	
		Misters	
		Other	
		**Water main disruptions**	
		Opportunity for water main breakage	
		Length of shut down in hours/days	
		Off-site construction of municipal water delivery system	
		**Central Utility Plant Modifications/Alterations**	
		Underground utility connections	
		**Cooling Towers**	
		Replacement	
		Addition	
		**Disinfection of underground utility connections or building**	
		Does the project call for building water main disinfection?	
		When during the project is this activity scheduled to be performed?	

SUPPLEMENT S2 - WMC-ICRA PRECONSTRUCTION RISK ASSESSMENT CHECKLIST (page 2 of 2)

YES	NO	BUILDING WMC RISK FACTORS	NOTES SECTION
		**High Water Age / Stagnation Challenges (Circle One)**	
		How long will building water system experience dormancy or shut-downs?	
		< 24 hours; ≤ 7 calendar days; ≤ 30 calendar days; > 30 calendar days	
		**Inadequate residual disinfectant**	
		Is disinfectant residual measurement between TRO > 0.5 ppm or < 4.0 ppm / or / FRO > .02 ppm or < 4.0 ppm?	
		**Provide verification of existing residual disinfectant measurements**	
		Incoming municipal water main	
		Pre - Post water softener	
		Pre - Post RO or other central filtration system	
		Return hot water loop system	
		Distal distribution points on each floor of construction	
		**Confirm existing temperature control ranges**	
		Hot water storage temperature	
		Hot water range (per WMP at fixture delivery)	
		Cold water range (per WMP)	
		Does the project utilize point-of-use mixing values?	
		**Unoccupied areas or low or no use areas pre or post occupancy**	
		Shell areas with water in piping system	
		Unoccupied areas with water in piping system	
		Low use areas with water in piping system	
		**Vibration activities**	
		Demolition	
		Jackhammering	
		Saw cutting	
		What departments are above, below, or downstream/near vibration activities?	
		**Efficiency design challenges**	
		Water system design for conservation measures	
		Participating in LEED, WELL, or FitWEL building rating systems	
		Auto-fixtures (electronic, sensor, or push button - ex. surgical scrub sinks)	
		Aerators	
		Ligature-resistant fixtures (i.e., behavioral health, security fixtures)	
		Other - mixed temperature fixtures, etc.	
		**Repressurization (start-up and shut-down)**	
		Will any part of the building water system experience re-pressurization?	
		**Building/Plumbing Water service disruption**	
		New construction tie-ins	
		Replacement valves	
		Meters	
		Pumping failures	
		Other system repairs or component replacement	
		Emergency conditions	
		**Construction equipment with water reservoirs typically with spray activities**	
		Showers	
		Spray nozzles	
		Misters	
		Other	
		**Disinfection of building water distribution system**	
		Does the project call for building water distribution system disinfection?	
		Have ports and isolation valves been installed for this section of the building?	
		When during the project is this activity scheduled to be performed?	
		**Central Utility System Modifications/Alterations**	
		Water heaters	
		Heat exchangers	
		Water storage	
		Hot water loop system	
		Boiler system	
		Other central building water system components	

Citation: Scanlon MM, Gordon JL, Tonozzi AA, and Griffin SC (2022). Reducing the Risk of Healthcare Associated Infections from *Legionella* and Other Waterborne Pathogens Using a Water Management for Construction (WMC) Infection Control Risk Assessment (ICRA) Tool. *Infectious Disease Reports* , 14 (3) Supplement S2.
